# Effects of *Myroxylon pereirae*, Phenytoin, and Clinoptilolite After Pharyngocutaneous Fistula: An Experimental Animal Model

**DOI:** 10.1002/ohn.1179

**Published:** 2025-02-21

**Authors:** Erim Pamuk, Enes Dogan, Olcay Kurtulan, Yeşim Gaye Güler Tezel, A. Arzu Yiğit

**Affiliations:** ^1^ Department of Otorhinolaryngology Hacettepe University Ankara Turkey; ^2^ Department of Pathology Hacettepe University Ankara Turkey; ^3^ Department of Physiology Başkent University Ankara Turkey

**Keywords:** balsam of Peru, clinoptilolite, *Myroxylon pereirae*, pharyngocutaneous fistula, phenytoin

## Abstract

**Objective:**

The objective of this study was to conduct a comparative evaluation of the effects of *Myroxylon pereirae* (MP), phenytoin, and clinoptilolite on wound healing in an experimental animal model for the treatment of pharyngocutaneous fistula (PCF).

**Study Design:**

Prospective controlled animal study.

**Setting:**

Animal laboratory.

**Methods:**

Forty‐four male Sprague–Dawley rats were randomly assigned to one of four groups: sham control group, MP group, phenytoin group, and clinoptilolite group. A PCF was created in each rat via surgical intervention, followed by a course of topical treatment administered twice daily for a period of 7 days. The healing of the fistula was evaluated both macroscopically and histopathologically.

**Results:**

Macroscopic fistulae developed in 90% of the control group, 18% of the MP group, and 27% of the phenytoin group (*P* = .005). The phenytoin group had the lowest inflammation scores, which were significantly lower than the clinoptilolite and control groups (*P* = .006 and *P* = .001). The MP group had the highest levels of fibroblast proliferation and collagen accumulation (*P* < .001 and *P* = .001, respectively). The level of inflammation and amount of fibroblast proliferation, angiogenesis, and collagen accumulation in the clinoptilolite group was lower than in the control group, but none of these differences were significant statistically.

**Conclusion:**

MP and phenytoin improved the healing of PCF, particularly by reducing the inflammation and promoting the of fibroblast proliferation and collagen accumulation. Clinoptilolite did not demonstrate a notable advantage in any of these parameters. These findings suggest that MP and phenytoin may serve as potential agents in the management of PCF.

The formation of a pharyngocutaneous fistula (PCF) represents a significant postoperative complication in patients who have undergone head and neck surgery, particularly in those who have undergone laryngectomy. The incidence of PCF following primary total laryngectomy is highly variable, with reported rates ranging from 8% to 65%.^1^ Despite the implementation of prophylactic measures throughout the perioperative period, the occurrence of PCF cannot be entirely ruled out in some subsets of patients. In case of such occurrence, a range of conservative and surgical treatment options is available. The conservative management of PCF, which includes local wound care, debridement, pressure dressings, broad‐spectrum antibiotics, non‐oral feeding and intravenous nutrition, has demonstrated success rates of 70%–80% for PCF closure.^2^ However, prior radiotherapy, malnutrition, or surgical scarring can impair the wound healing process. Negative‐pressure vacuum‐assisted wound therapy, hyperbaric oxygen therapy, and botulinum toxin injections may facilitate the healing of PCF.^3^, ^4^ Regional/free flaps can also be employed in the treatment of large, high‐risk, or refractory PCF.^5^, ^6^ The principal objective of local wound care in the management of PCF is to facilitate the formation of granulation tissue and closure of the tract. This is achieved by creating an aseptic wound environment and stimulating epithelial and fibrous tissue growth.

*Myroxylon pereirae* (MP) (balsam of Peru), a natural resin derived from a tropical tree native to Central and South America, has been employed in the treatment of a diverse array of dermatological conditions, including poorly healing wounds, decubitus, eczema, pruritus, hemorrhoids and anal pruritus, due to its antiseptic, anti‐inflammatory and wound‐healing properties.^7^, ^8^ It is a mixture of different compounds, the major ingredients of which are benzyl benzoate, tridecanoic acid, and cinnamic acid. These are known for their antimicrobial and antioxidant properties.^9^, ^10^ It has been demonstrated that MP, when combined with castor oil and trypsin, accelerates the healing process of skin graft donor sites and reduces the risk of infection.^8^ Nevertheless, the efficacy of this treatment on a specific wound type, such as PCF, which are subjected to continuous salivary flow, has yet to be evaluated.

Phenytoin, in addition to its established use as a pharmaceutical agent for the treatment of epilepsy, has also been the subject of research into its potential beneficial effects on wound healing. It has been demonstrated that the topical use of phenytoin may facilitate the wound healing process in diabetic ulcers and nasal septal perforations due to its anti‐inflammatory, antibacterial, analgesic, and cellular proliferation‐promoting properties.^11^, ^12^, ^13^ These data indicate that phenytoin may have a potential therapeutic role beyond its traditional uses.

Clinoptilolite, a natural zeolite mineral occurring in volcanic rocks, has attracted attention as a potential therapeutic agent in wound healing. This mineral is regarded as a potentially valuable component in the management of wounds, particularly due to its antimicrobial properties, cationic change ability to swap heavy metal ions in the environment with its own sodium, calcium, magnesium and potassium ions, and the capacity to absorb exudate in an exothermic reaction that enhances coagulation factors.^14^ It has been proposed that it may be a useful agent with anti‐inflammatory and antibacterial effects, which promote its use in wound management.^15^, ^16^

The objective of this study was to demonstrate and compare the efficacy of MP, phenytoin and clinoptilolite, in the treatment of PCF in an experimental animal model.

## Materials and Methods

### Animals and Study Design

This study complies with the ARRIVE guidelines and carried out in accordance with EU Directive 2010/63/EU for animal experiments. It was approved by Başkent University Ethical Committee for Experimental Research on Animals (Project no: DA24/01, date: 29.1.2024) and supported by Başkent University Research Fund. A priori power analysis was conducted using G*Power version 3.1.9.4 for estimation of the required sample size based on data from Liu et al.^17^ With a significance criterion of *α* = .05, power = .80 and medium effect size = .58, the minimum sample size was determined to be 40 animals. In addition, four animals were included to allow for any potential losses that might occur during the course of the study. The study was designed and conducted in accordance with the Animal Experimentation Regulations and the 3R principles.

The study employed a total of 44 male Sprague–Dawley rats with an average weight of 250–300 g. The rats were provided from the Animal Production and Research Center of Baskent University, and all the research procedures were carried out at the same center. The animals were housed in accordance with standard conditions and 12/12 light/dark cycle, and had access to food and water ad libitum. The rats were randomly assigned to one of four groups: the MP group (*n* = 11), the phenytoin group (*n* = 11), the clinoptilolite group (*n* = 11), and the sham control group (*n* = 11). Two rats died during the course of the study, one from the clinoptilolite group and one from the sham control group.

### Surgical Procedure

A PCF was successfully created in all rats. To achieve the optimal anesthetic effect, a dosage of 7 mg/kg xylazine and 60 mg/kg ketamine was administered intraperitoneally. Once deep anesthesia had been achieved, the neck area was shaved and the animals were positioned in a supine position, with neck extension achieved with the assistance of a small roll of gas. The neck was cleansed with a povidone‐iodine solution, and a sterile drape was applied. A vertical incision of approximately 1.5 cm in length was made on the midline of the neck.

**Figure 1:**
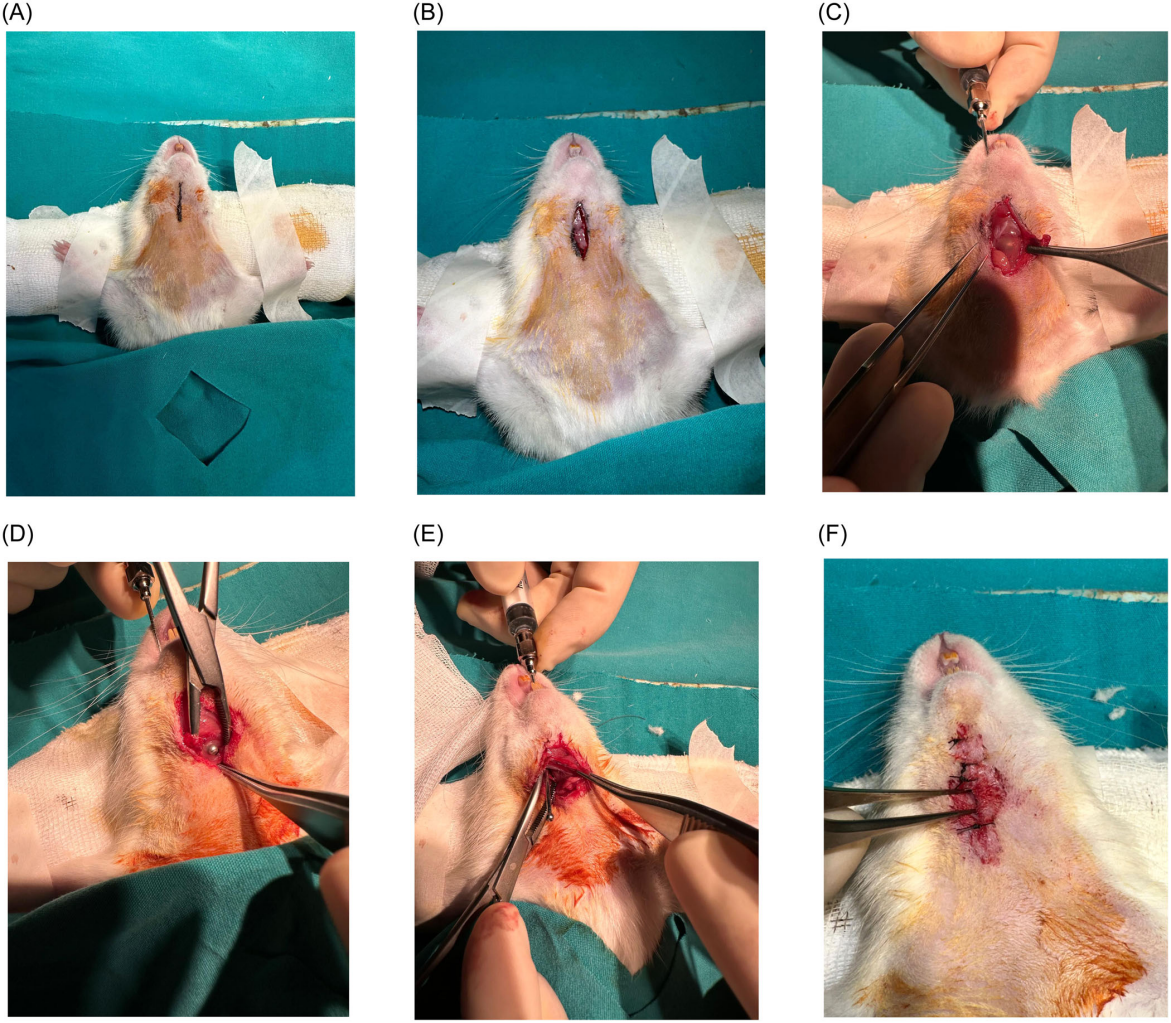
Steps of pharyngocutaneous fistula formation: (A) Neck preparation. (B) Midline neck incision. (C) Localization via gavage needle. (D) Incision at needle site. (E) Pharyngotomy widening. (F) Skin closure, leaving the middle open.

Once the subcutaneous tissues had been transected a gavage cannula was inserted into the mouth in a controlled manner in order to facilitate the visualization of the pharyngotomy point. Subsequently, a longitudinal incision measuring 1 cm was made with a surgical blade. Subsequently, the skin incision was sutured with two 5.0 silk sutures in a loose manner at the superior and inferior margins of the incision, while the mid portion of the incision and the underlying pharyngotomy site were left unsutured (Figure 1).

### Postoperative Management and Topical Application of Study Agents

**Figure 2:**
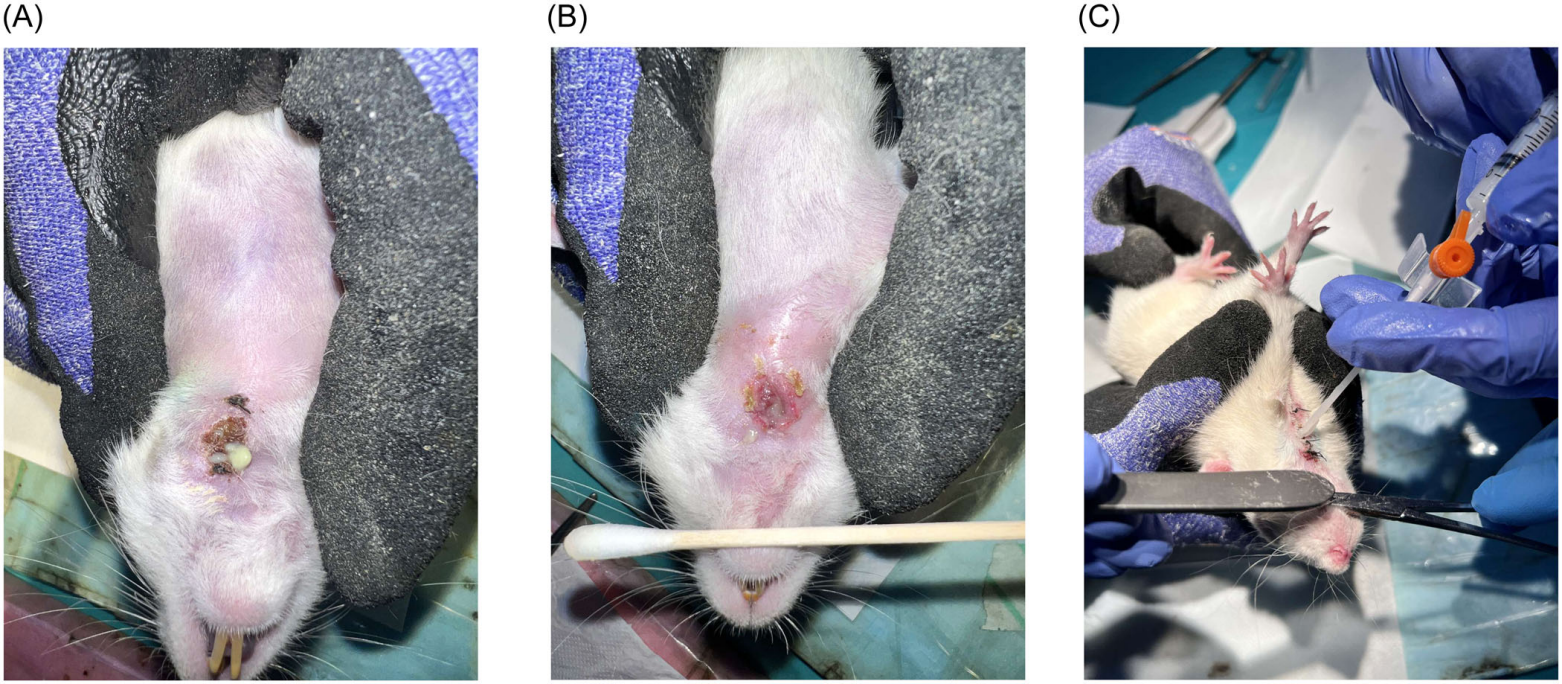
Wound care of the pharyngocutaneous fistula. (A) Fistula tract covered with granulation tissue. (B) Active purulent discharge from the fistula tract. (C) Application of topical treatment using an intravenous catheter.

Following surgical intervention, the oral intake of all animals was meticulously monitored on a regular basis. In case of a notable reduction in oral intake of feed, 5% dextrose was administered intraperitoneally as a supplement. No botulinum toxin injection was applied to salivary glands. Previous studies have indicated that three days is the average time for the development of PCF.^17^, ^18^ Therefore, the animals were observed for a period of three days following the surgical procedure, during which no treatment was administered. On the 3rd day of the study, topical administration of MP resin, phenytoin (tablets ground to prepare a powder, Epanutin®, Pfizer) and clinoptilolite (Toxapravent Skin Powder, Froximun®) was applied at an interval of twice daily (Figure 2). The test agents were applied in a mixture with petroleum jelly (Vaseline®, Unilever). The sham control group received only a daily application of petroleum jelly. At the conclusion of the seven‐day treatment period (10th day postoperatively), all animals were euthanised by cervical dislocation, following a macroscopic evaluation of the wound site (Figure 2). PCF was defined as absent if there was no mucosal disruption and the pharyngotomy site had completely healed. Conversely, a fistula was defined as present if the pharyngotomy site remained open and food residue was observed at the site.^19^

### Pathological Evaluation

**Figure 3:**
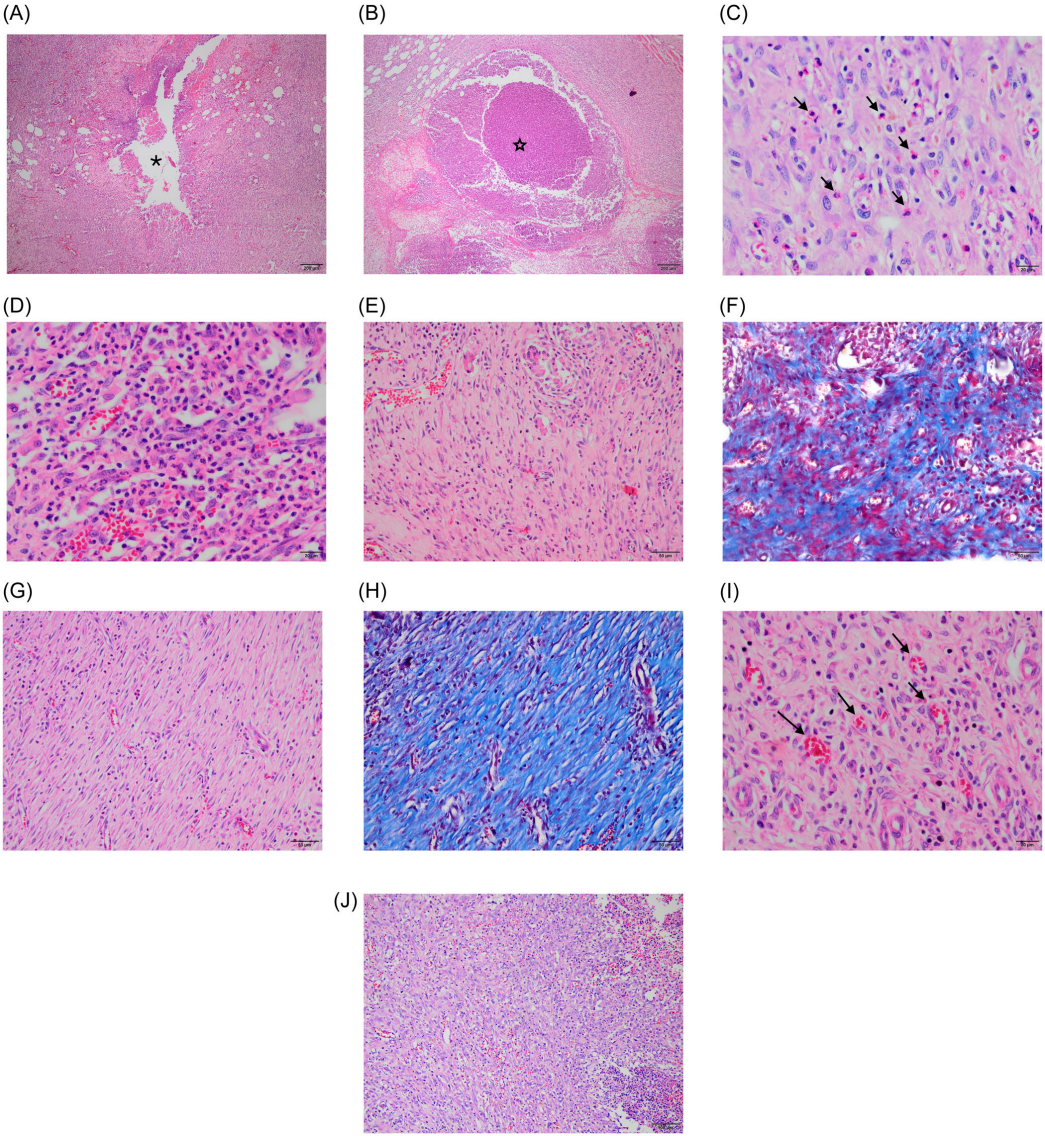
Histopathological evaluation of fistula tract: (A, B) Inflammatory reaction, exudate‐filled lumen (asteriks shows fistula lumen). (C, D) Inflammatory cells (grades 2 and 4) (arrows show inflammatory cells). (E–H) Collagen/fibrosis (grades 2 and 4). (I, J) Neovascularization (grade 2) and fibroblast proliferation (grade 4) (arrows show vessels).

Following the decapitation of all animals in the control and treatment groups, the specimens were stored in containers containing a 10% formaldehyde solution for a period of 24 h. Subsequently, sections including the skin, subcutaneous tissues, pharynx, and fistula tract were taken for pathological examination. The tissue was embedded in paraffin, sectioned 3–4 μm thickness using a rotary mirotome, sections were taken on glass slides and stained with haemotoxylin & eosin (HE) and Masson's trichrome (MT). The histopathological examinations were conducted by two pathologists, and scoring was performed independently. The pathologists were blinded to the group assignments of each specimen to avoid bias. Any discrepancies between the two pathologists were resolved through discussion. The 0–4 Ehrlich and Hunt classification system was employed for the purpose of scoring, as previously described.^17^, ^20^, ^21^ The microscopic evaluation of the pathological examination included an assessment of the following parameters: inflammation, fibroblast proliferation, angiogenesis and collagen accumulation. The parameters were scored on a scale of 0–4 as follows: The scoring system employs the following categories: 0 = no evidence; 1 = occasional evidence; 2 = light scattering; 3 = abundant evidence; 4 = confluent cells or fibers. For evaluation of angiogenesis (neovascularization) number of vessels in one high power field (×400) was counted and scored as follows: 1: 1–5, 2: 6−10, 3: 11–20, 4: >20 (Figure 3).

### Statistical Analysis

Statistical analyses were conducted using IBM SPSS Statistics for Windows v.23.0 (IBM Corp.). Categorical variables were investigated employing the *χ*² test and Fisher's exact test for small‐sample data (*n* < 5). Student's *t*‐test was employed for the purpose of comparing the means of normally distributed independent variables. Kruskal–Wallis test was employed for nonnormally distributed independent variables in multiple groups. In cases where significant differences between groups were identified through the Kruskal–Wallis test, individual groups were subsequently compared using the Mann–Whitney *U* test. Following the application of Bonferroni correction for multiple comparisons, a value of *P* < .05/6 = .0083 was deemed statistically significant in pairwise group comparisons using the Mann–Whitney *U* test. The level of statistical significance was set at *P* < .05, and all reported *P* values are two‐sided. DeepL was used for language editing.

## Results

A total of 42 male Sprague–Dawley rats were included for the study analysis. The mean weight of the rats was 228 ± 46 g. No statistically significant difference was observed between the groups in terms of rat weight (*P* = .117). The histopathological parameters of inflammation, fibroblast proliferation, and collagen accumulation exhibited notable differences between the groups, whereas no significant discrepancy was observed in terms of angiogenesis (*P *= .001, *P *< .001, *P *= .001, and *P *= .081, respectively) (Table 1).

**Table 1:** Histopathological scores, fistula rates and weight among study groups

	*Myroxylon pereirae* (*n* = 11)	Phenytoin (*n* = 11)	Clinoptilolite (*n* = 10)	Sham control (*n* = 10)	*P*‐value*
Inflammation	2.45 ± 0.93	1.82 ± 0.98^a^	3.1 ± 0.73^a^	3.56 ± 0.52^a^	**.001**
Fibroblast proliferation	3.36 ± 0.67^b^	3 ± 1^b^	1.9 ± 0.87	1.67 ± 0.7^b^	**<.001**
Angiogenesis	3.27 ± 0.78	2.91 ± 0.94	3 ± 1.05	2.2 ± 0.7	.081
Collagen accumulation	3.55 ± 0.52^c^	3.45 ± 0.68^c^	2.6 ± 1.07	1.89 ± 0.78^c^	**.001**
Weight (g)	217 ± 32	231 ± 23	231 ± 27	234 ± 87.4	.117
Gross fistula formation (yes/no)	2/9 (18%)^d^	3/8 (27%)^d^	5/5 (50%)	9/1 (90%)^d^	**.005**

No difference were observed in angiogenesis scores between any of the study agents. Bold values indicate statistical significant at *P* < .05.

* Bonferroni adjustment was applied for pairwise comparisons of groups. *P* < .0083 was accepted as statistically significant for the pairwise Mann–Whitney *U* test.

^a^ There was a significant difference between phenytoin, and clinoptilolite and sham control groups (*P* = .006 and *P* = .001, respectively).

^b^ There was a significant difference between sham control group, and *Myroxylon pereirae* and phenytoin groups (*P* < .001 and *P* = .007, respectively).

^c^ There was a significant difference between sham control group, and *Myroxylon pereirae* and phenytoin groups (*P* < .001 and *P* = .001, respectively).

^d^ There was a significant difference between sham control group, and *Myroxylon pereirae* and phenytoin groups (*P* < .001 and *P* = .003, respectively).

**Figure 4:**
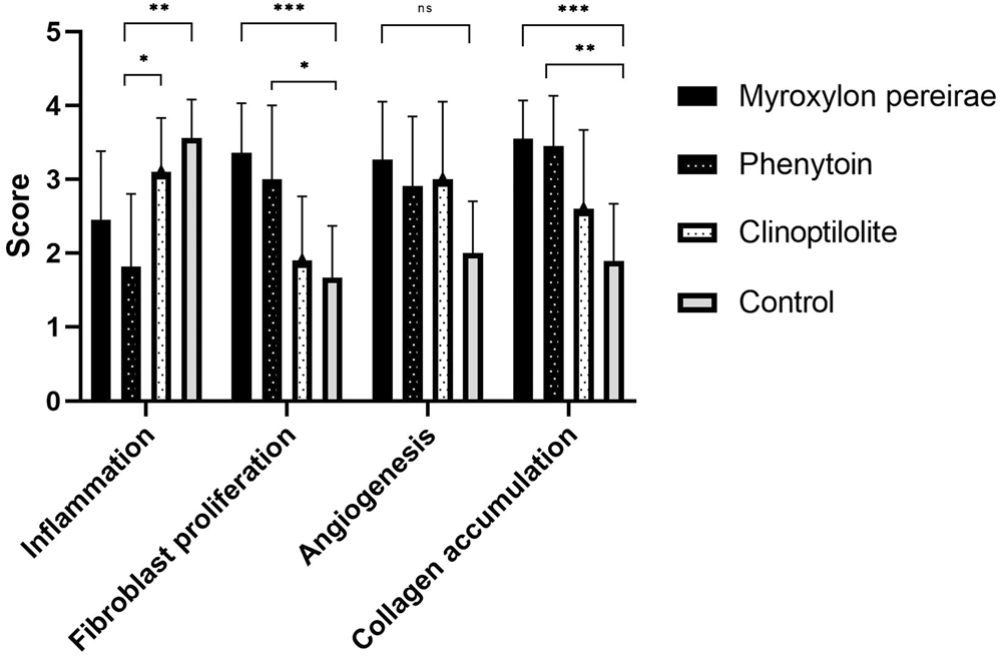
Graphical representation of histopathological scores according to different topical agents. *P < 0.008; **P < 0.005; ***P < 0.001.

A pairwise comparison of the groups revealed that the lowest inflammation value was observed in the phenytoin group, which exhibited a significantly lower level of inflammation than both the clinoptilolite and control groups (*P* = .006 and *P* = .001, respectively). Although the inflammation value was also lower in the MP group in comparison to the control group, the observed difference did not reach the level of statistical significance (*P* = .01). The highest level of fibroblast proliferation was observed in the MP group. The fibroblast values observed in both the MP and phenytoin groups were significantly higher than those observed in the control group. Nevertheless, no statistically significant difference was identified between the two groups (*P* = .478). Furthermore, the highest value for collagen accumulation was observed in the MP group. The groups treated with phenytoin and MP exhibited significantly elevated values in comparison to the control group; however, no notable discrepancy was evident between these two groups (*P* = .898). Macroscopically, fistula formation was observed in 90% of the control group, 18% in the MP group and 27% in the phenytoin group (*P* = .005). No significant difference was identified between the groups with respect to angiogenesis. Despite the clinoptilolite group exhibiting reduced inflammation and enhanced fibroblast proliferation, as well as increased angiogenesis and collagen accumulation in comparison to the control group, no notable discrepancy was identified in any of the parameters. Figure 4 presents a graphical representation of the scores of the study variables with respect to each group.

## Discussion

A review of the literature reveals a relatively limited number of studies that have employed the experimental animal model of PCF. In 1993, Weiss et al. pioneered the creation of PCF in an experimental animal model, utilizing white rabbits and proposing a method to excise the mid portion of the thyrohyoid membrane.^22^ Despite the well‐defined methodology outlined in their study, it was not feasible for us to fully adopt it in the current investigation due to the necessity of a high number of animals, which was not possible with rabbits. In 2008, Liu et al. conducted an investigation into the effects of different suture materials on pharyngeal closure in a rat model. A vertical incision was created from the hyoid to the cricoid cartilage on the lateral border of the pharynx to create a pharyngeal wound. The results demonstrated that 6‐0 vicryl followed by fibrin glue exhibited the lowest fistula rates and the highest inflammatory cell infiltration, neovascularisation, fibroblast activity, and collagen deposition.^17^ The manner in which the pharynx and skin are sutured during the creation of the PCF exhibits inconsistency across the studies. In a subsequent study, Liu and colleagues tested the effects of oxidized regenerated cellulose on pharyngeal wounds in a similar rat model.^18^ In both studies, the pharynx mucosal incision was sutured when creating a PCF.^17^, ^18^ However, as in our own study, Demir and colleagues left the pharyngeal mucosa open to allow for the formation of a fistula, and only sutured the skin incision.

All of the studies included in the literature are based on the application of the test substance or method at the initial PCF formation session. However, in our study, the topical treatment agents were applied on a daily basis and continuously to the PCF area, in accordance with the similar clinical practice. In light of the aforementioned considerations, the timing of the commencement of the topical treatment application was not predetermined. Nevertheless, it has been demonstrated by Liu et al. that the inflammatory response reaches its highest level three days after the procedure.^17^, ^18^ In light of this finding and in order to emulate the delayed onset of PCF in patients, we opted to begin the application of the topical treatment on the 3rd day. The day of sacrifice of the animals also varies across the studies. Some authors sacrificed at the postoperative 7th day, whereas, similar to our study, some others sacrificed at the 10th day.^17^, ^18^, ^19^, ^20^ Furthermore, there are studies that euthanised even at the postoperative 14th day.^23^

MP resin has a long history of use in medicine, particularly as an antiseptic and wound healing accelerator among South American natives, both in the pre‐Columbian era and in the present day.^8^ Furthermore, it has emerged as an alternative tool that can be used as a marker for fragrance sensitivity, contact dermatitis and as a treatment agent in scabies.^7^, ^24^, ^25^ The effects of MP resin on wound healing have been understudied in the literature, with the majority of studies conducted in the 2000s. In 2003, Carson et al. observed that the use of an ointment consisting of MP resin combined with castor oil and trypsin in patients with chronic wounds resulted in improved healing at donor sites for split‐thickness skin grafts.^8^ Additionally, there are retrospective case series and case reports that have been published on this topic. Topical ointment therapies have been shown to improve healing in patients with chronic wounds when MP is included.^26^, ^27^, ^28^ The continuous saliva flow, which impairs the normal wound healing process, is the unique feature of PCF, which makes it a distinct type of wound from those located at other localizations of the body. The MP‐treated PCF group exhibited the highest amount of fibroblast proliferation, angiogenesis, and collagen accumulation. Furthermore, the lowest rate of PCF at the conclusion of the study was also observed in the MP resin‐treated group. Given the saliva flow which transports intraoral bacteria to the wound site, the lower inflammatory response may be attributed to the strong antiseptic features of MP. The rapid epithelialisation and low inflammatory response observed in the MP group suggest that this substance may play a role in the wound healing process. However, the potential for allergic reactions should be kept in mind when using the agent in a clinical setting.

The use of phenytoin in the management of PCF is regarded as a promising avenue of investigation due to its antimicrobial and cellular proliferation‐promoting properties. In 1996, Anstead et al. proposed that phenytoin accelerates the formation of granulation tissue by enhancing fibroblastic activity, thereby markedly enhancing wound healing.^29^ A review by Keppel Hesselink demonstrated that phenytoin enhances collagen synthesis by stimulating fibroblast proliferation, thereby accelerating granulation tissue formation.^11^ A recent systematic review by Sadiq et al. reported that topical phenytoin enhances wound healing and offers analgesic and antibacterial properties with minimal adverse effects.^13^ The review included three studies which reported improved wound healing in oral biopsy wounds, but found no studies on PCF. Sadiq et al. emphasized the necessity of further studies on optimal dosage, frequency and delivery vehicles.^13^ Our study demonstrated that phenytoin exhibited the most pronounced anti‐inflammatory effect among the tested substances. Furthermore, the angiogenetic, fibroblast proliferating, and collagen accumulating effects were markedly increased in comparison to the control group. The findings indicate that the utilization of phenytoin may also represent a promising avenue in the wound healing process of PCF.

Clinoptilolite is still regarded as a relatively novel agent in the context of wound management. It is hypothesized that it can facilitate wound healing due to its anti‐inflammatory, oxygen reservoir and exudate‐absorbing properties.^30^ In 2012, Li et al. demonstrated improved wound healing effects of natural clinoptilolite in a rabbit model with complex joint injury.^31^ Çelikbaş et al. reported that clinoptilolite can contribute to the healing of extraction wounds and bone formation in their diabetic rat model.^32^ However, there are also reports which failed to demonstrate any significant effect of clinoptilolite in wound healing. Samadian et al. proposed that, despite the potential of zeolite to load antibiotics or other effective compounds, there were no significant benefits of adding clinoptilolite to standard alginate hydrogel in wound management, in terms of re‐epithelialisation, granulation tissue thickness, collagenisation, inflammatory cell recruitment, and angiogenesis. In 2022, Deinsberger et al. reported that there were no significant differences in wound healing or wound conditions between the clinoptilolite‐treated group and the standard of care‐treated control group in their randomized phase I clinical trial.^15^ Similarly, no significant differences were observed in the pathological features or fistula rate between the clinoptilolite‐treated and control groups in our study. Additionally, there is a paucity of literature comparing the effects of clinoptilolite on PCF healing.

The small sample size of this study was a limitation that may reduce the statistical power. Additionally, the suturing methods used to create PCF and the evaluation methods for macroscopic PCF vary among studies in the literature.^17^, ^18^, ^19^, ^23^ This variability also presents a limitation in confidently comparing the results across studies. The inconsistency in the existing literature underscores the need for further research on wound healing in PCF.

## Conclusion

The results demonstrated that MP and phenytoin exhibited a notable therapeutic impact on the management of PCF, particularly in the reduction of inflammation and the promotion of fibroblast proliferation and collagen accumulation. The application of clinoptilolite did not result in a notable improvement in these parameters. The results indicate that MP and phenytoin may represent promising agents for the treatment of wound healing. Further evidence on human subjects is required to document the efficacy and potential side effects.

## Author Contributions

**Erim Pamuk**, design, analysis, interpretation of data for the work, drafting, final approval, final agreement. **Enes Dogan**, design, interpretation of data for the work, critical revision, final approval, final agreement. **Olcay Kurtulan**, acquisition and interpretation of data for the work, critical revision, final approval, final agreement. **Yeşim Gaye Güler Tezel**, acquisition and interpretation of data for the work, critical revision, final approval, final agreement. **A. Arzu Yiğit**, conception, acquisition of data for work, critical revision, final approval, final agreement.

## Disclosure

### Competing interests

None.

### Funding

This study was funded by Başkent University Research Fund.
